# Effects of remote Baduanjin Qigong intervention on quality of life and physical function in patients with mild to moderate Parkinson’s disease: a protocol of randomized controlled trial

**DOI:** 10.3389/fneur.2025.1620424

**Published:** 2025-08-06

**Authors:** Jizhe Yu, Yi Jia, Guanglin Ma, Dong Wang, Zhen Zhang, Zengbao An, Yingkui Li

**Affiliations:** ^1^China National College of Martial Arts, Beijing Sports University, Beijing, China; ^2^Department of Physical Education, Beijing Polytechnic College, Beijing, China

**Keywords:** Parkinson’s disease, Baduanjin Qigong, remote intervention, physical function, quality of life

## Abstract

**Background:**

This study aims to evaluate the effects of remote Baduanjin Qigong intervention on the quality of life and physical function in patients with mild to moderate Parkinson’s disease (PD) through a randomized controlled trial (RCT) and to compare its efficacy with conventional exercise rehabilitation.

**Methods/design:**

This RCT will enroll participants who, following baseline assessments and physical function tests, will be randomly allocated into one of three groups: the Qigong group (QG), the conventional exercise rehabilitation group (EG), or the control group (CG). The QG will engage in live-streamed Baduanjin (Eight-Section Brocade) sessions three times per week, each lasting 40 min, with additional self-practice encouraged. The EG will undergo a structured regimen consisting of moderate period high-intensity resistance training (MP-HI-RT) combined with moderate period low-intensity aerobic exercise (MP-LI-AE), also performed remotely in three 40-min sessions per week. The CG will continue their routine daily activities without additional exercise intervention. Outcome assessments will be conducted at baseline (Week 1), mid-intervention (Week 6), and post-intervention (Week 12) to evaluate both short-term and long-term effects. The primary outcomes include quality of life, evaluated using the Parkinson’s Disease Questionnaire-39 (PDQ-39), the MOS 36-item Short Form Health Survey (SF-36), and the Unified Parkinson’s Disease Rating Scale developed by the Movement Disorder Society Part I (MDS-UPDRS Part I). Secondary outcomes encompass reaction time, balance, physical coordination, flexibility, and walking capacity.

**Discussion:**

The importance of exercise rehabilitation for PD is increasingly recognized by doctors and patients. However, accessible and practical rehabilitation methods remain an area of active investigation. Remote Baduanjin Qigong intervention via the Internet offers a promising alternative for mild to moderate Parkinson’s disease patients.

**Clinical trial registration:**

https://www.chictr.org.cn/bin/project/edit?pid=268557, Identifier ChiCTR2500101461.

## 1 Background

Parkinson’s disease (PD) is the fastest-growing age-related neurodegenerative disorder worldwide and ranks as the second most prevalent neurological condition after Alzheimer’s disease. Affecting more than six million individuals globally, PD significantly compromises quality of life, impacting both physical and mental well-being (1, 2). Without effective intervention, PD patients experience significant impairments in motor function, leading to a substantial decline in quality of life, characterized by symptoms such as sleep disturbances, speech impairments, and emotional disorders (3, 4). Although pharmacological treatment remains the primary approach for alleviating PD symptoms, its clinical application is often constrained by side effects, the development of drug resistance, high costs, and so on (5). In contrast, physical exercise, which is free from adverse effects, has demonstrated positive outcomes in the treatment of other neurodegenerative diseases (6–8). Consequently, increasing research has focused on exercise interventions as a non-pharmacological approach to mitigating PD symptoms.

Existing research has demonstrated that exercise interventions can alleviate key clinical symptoms of PD, including postural instability, muscle rigidity, tremors, and bradykinesia. Moreover, these interventions contribute to improvements in physical function, enhancing muscle strength and balance, while also exerting positive effects on non-motor symptoms such as mood and cognitive function (9–13). As a result, exercise interventions are now widely recognized as an integral component of comprehensive PD management (14, 15). However, conventional exercise interventions are often limited by factors such as transportation barriers (e.g., long travel distances, lack of accessible public transportation), time constraints and high costs, making it challenging for many patients to access professional exercise guidance and rehabilitation. In recent years, with the rapid advancement of modern internet technology, remote interventions for PD patients have emerged as a feasible solution (16–18).

Baduanjin Qigong is a mind–body exercise that integrates mental regulation, breathing techniques, and physical movement. It is developed based on ancient Chinese Qigong literature and must be certified by the Chinese Health Qigong Association (19). Previous research has shown that Baduanjin Qigong training not only strengthens core muscle groups and improves postural stability in PD patients but also alleviates anxiety, depression, and sleep disturbances, thereby significantly enhancing their quality of life (20–22). However, studies investigating the effects of remote Baduanjin Qigong interventions on symptom improvement in patients with mild to moderate PD remain limited. Furthermore, no conclusive evidence has established whether Baduanjin Qigong offers superior benefits compared to traditional exercise interventions, such as combined aerobic exercise and resistance training. Therefore, this study seeks to assess the effects of remote Baduanjin Qigong intervention on the quality of life and physical function of patients with mild to moderate PD through a randomized controlled trial (RCT) with comparative analysis.

## 2 Objectives

1. To assess the feasibility and effectiveness of Internet-based remote intervention as a practical approach for exercise rehabilitation in individuals with PD.
2. To determine whether remote Baduanjin Qigong intervention enhances quality of life and physical function in patients with mild to moderate PD.
3. To compare the therapeutic effects of remote Baduanjin Qigong intervention with those of conventional exercise rehabilitation (aerobic and resistance training) in alleviating clinical symptoms and improving functional outcomes in PD patients.

## 3 Design and methods

### 3.1 Design

**Figure 1:**
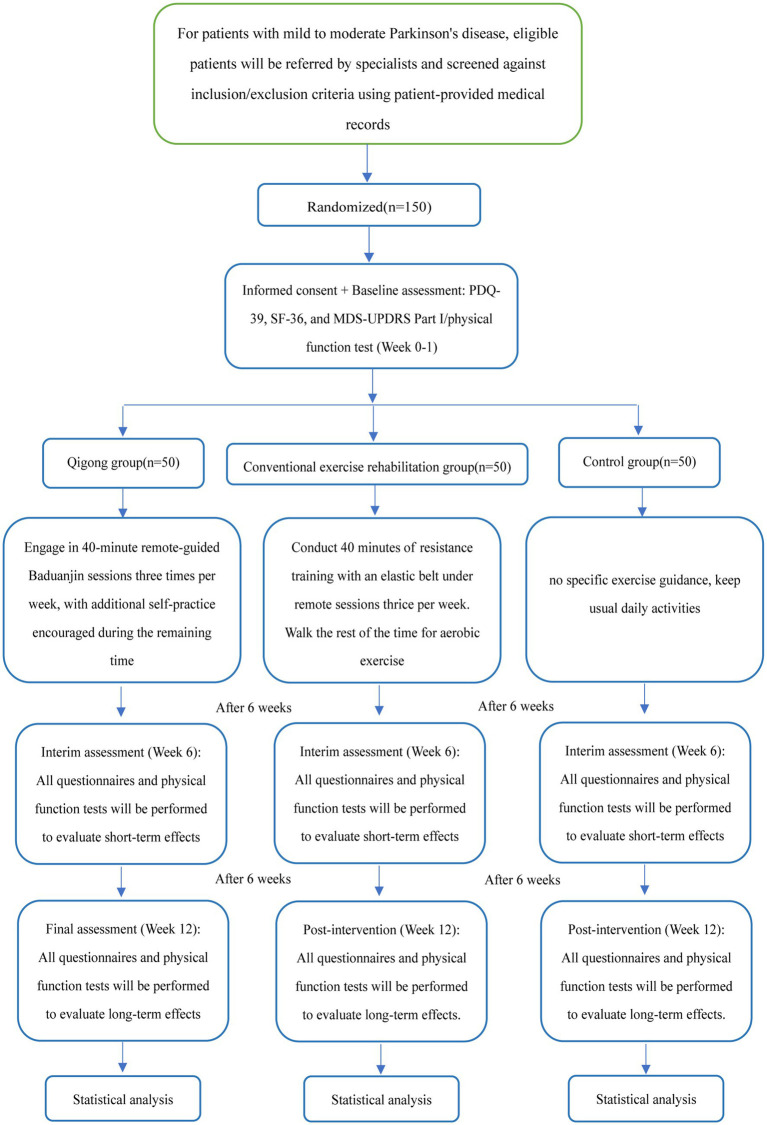
The flow chart of the trial design.

This study is a 12-week randomized controlled trial (RCT). Participant recruitment will be initiated in June 2025, and data analysis will be conducted once the required sample size has been achieved. The study design flowchart is presented in Figure 1.

### 3.2 Subject recruitment

#### 3.2.1 Inclusion criteria

1. Diagnosis of PD based on the Hoehn & Yahr staging system (Stages I–II).
2. Male or female patients aged between 50 and 80 years.
3. Currently receiving stable anti-Parkinsonian medication, with no changes in the treatment regimen for at least 3 months.
4. Basic self-care ability with no severe cognitive impairment (Mini-Mental State Examination [MMSE] score ≥24).
5. Ability to follow simple instructions and absence of uncontrolled chronic diseases.
6. Able to safely walk independently for at least 500 feet without an assistive device.
7. No prior experience with Baduanjin Qigong practice and willingness to sign an informed consent form.

#### 3.2.2 Exclusion criteria

1. Prior experience with systematic Baduanjin Qigong training or resistance exercise programs.
2. Severe cardiovascular disease, musculoskeletal disorders, or other conditions that significantly impair physical activity.
3. Severe mental illness or cognitive dysfunction (MMSE score <24).
4. Recent deep brain stimulation (DBS) treatment.
5. Inability to access the internet for remote training.

#### 3.2.3 Dropout criteria

1. Disease progression during the study period (e.g., advancement to Hoehn & Yahr Stage III).
2. Serious adverse events (e.g., falls resulting in fractures).
3. Inability to continue the intervention for any reason or voluntary withdrawal from the study.

#### 3.2.4 Recruiting methods

This study aims to recruit 150 eligible participants from the Beijing region through referrals from neurologists and PD specialists at major tertiary hospitals and movement disorder clinics. Specialists will briefly introduce the study to potential patients and their families. If patients express interest, the research team at Beijing Sport University will then contact them directly by phone, email, or message to complete eligibility screening and obtain informed consent. Recruitment will also be supported by advertisements in local newspapers, television, and reputable online platforms. To minimise gender bias and ensure the generalisability of the findings, efforts will be made to recruit an approximately equal number of male and female participants (50/50 ratio) whenever possible. The recruitment process will follow these steps:

1. Develop a recruitment plan and confirm participant sources.
2. Screen interested patients based on inclusion and exclusion criteria, including review of medical records, diagnostic certificates, or other documentation issued by PD specialists at tertiary hospitals or movement disorder clinics, along with medical history information provided by the patient or their family.
3. Establish procedures for contacting participants, including preparing relevant data, videos, and images to facilitate participants’ understanding of the study objectives.
4. Develop procedures for obtaining informed consent, ensuring that participants or their legal guardians sign the consent form.

### 3.3 Subject grouping

All participants will receive a comprehensive explanation of the study’s objectives, procedures, potential benefits, and possible risks before the trial. They will voluntarily sign an informed consent form and will be informed that they can withdraw from the study at any stage without any adverse consequences. After completing the informed consent process, participants will be randomly assigned in a 1:1:1 ratio to one of three groups—the Qigong group (QG), the conventional exercise rehabilitation group (EG), or the control group (CG)—using a computer-generated random number table. Participants in both the Baduanjin Qigong and conventional exercise rehabilitation groups will receive three remote live training sessions per week and will be encouraged to perform an additional two to three self-guided sessions weekly. Participants in the control group will maintain their usual daily activities without additional exercise intervention, but will be required to complete regular follow-ups and assessments.

### 3.4 Allocation

#### 3.4.1 Allocation concealment mechanism

To prevent selection bias, the researcher responsible for generating the randomization sequence will be independent from those determining participant eligibility. The randomized sequence will be generated using a computer-generated block randomization (SPSS 26.0) and will be stored by a research manager who will not be involved in the intervention or assessment process. Each allocation will be sequentially numbered and placed in an opaque, tamper-proof, and light-tight envelope. These envelopes will be securely sealed and stored by an independent research coordinator. Only after an eligible participant had completed baseline assessments and formally agreed to participate will the next envelope in sequence be opened by the research coordinator to reveal the intervention assignment. Neither the participants nor the interventionists will be aware of the allocation until the envelope is opened, ensuring strict allocation concealment.

### 3.5 Implementation

After completing baseline evaluations and physical function tests, participants will be randomized in a 1:1:1 ratio to one of the three groups: the Qigong group (QG), the conventional exercise rehabilitation group (EG), or the control group (CG). The research coordinator will open the next sealed envelope in sequence to reveal each participant’s assignment.

### 3.6 Qigong group (QG)

Participants in the Qigong group will practise Baduanjin (Eight-Section Brocade) through live-streamed video sessions under the guidance of professional instructors. Baduanjin is a traditional mind–body exercise that integrates physical movement, breath control, and psychological regulation. It consists of eight sequential movements, primarily involving upper and lower limb coordination, head–neck control, and spinal movement, all performed in synchronisation with rhythmic breathing. Each complete session will last approximately 10 to 15 min. During the first week, participants will receive systematic instruction sessions in small groups (five participants per group, with a total of ten groups) via a two-way live video conferencing platform. Certified Qigong instructors will guide participants step by step through the eight standard Baduanjin movements so that all participants can master the basic forms and key techniques. Instructors will closely monitor each participant’s postures, breathing techniques, and movement sequences, providing real-time corrections and movement modifications when needed. This real-time, two-way format will consistently be applied in all remote sessions during the intervention period to guarantee correct practice and optimize training effectiveness. From week 2 onward, participants will engage in three 40-min remote sessions per week, which included 5 min of warm-up, 30 min of core Baduanjin practice, and 5 min of cool-down exercises under professional supervision. Participants will also be encouraged to practice independently outside of the scheduled sessions to enhance training effectiveness. Participants who withdrew from the study will be asked to provide their reasons for discontinuation.

### 3.7 Conventional exercise rehabilitation group (EG)

Participants in the conventional exercise rehabilitation group will undergo a combined aerobic and resistance training intervention, following a structured moderate period high-intensity resistance training (MP-HI-RT) + moderate period low-intensity aerobic exercise (MP-LI-AE) (23). Participants will engage in three 40-min sessions per week, guided by professional two-way live video tutorials. Each session consisted of 5 min of warm-up, 30 min of core resistance training with resistance bands, and 5 min of cool-down exercises. The resistance load will be determined based on baseline muscle strength assessments to ensure appropriate intensity for each participant. Additionally, participants will be encouraged to engage in self-paced walking exercises outside of the supervised sessions, with walking intensity adjusted according to their pre-measured aerobic capacity. Participants who withdrew from the study will be asked to provide their reasons for discontinuation.

### 3.8 Control group (CG)

Participants in the control group will not receive specific exercise guidance and will be instructed to maintain their usual daily activities. Researchers will conduct regular monthly follow-ups to assess participants’ status and to confirm that they do not engage in additional exercise interventions during the study period.

### 3.9 Technology support and internet literacy

To ensure all participants can smoothly access the remote live sessions, brief internet literacy training will be provided before the start of the program. During this training, each participant’s ability to log in to the secure two-way video conferencing platform will be verified. If a participant is unable to operate the platform independently, their family members or caregivers will receive clear instructions and training to assist them. All sessions will be delivered via a two-way live format, and live session streams will be backed up to the cloud within the platform for internal monitoring purposes only.

In addition to the instructors, a designated research team member will be online throughout each live session to monitor technical stability and provide immediate assistance if needed. Participants will also have access to real-time technical support during class hours via instant messaging or phone contact, ensuring any network or access issues can be resolved promptly.

### 3.10 Adherence monitoring

To ensure participant compliance and maximize the effectiveness of the interventions, all participants in the Qigong group and the conventional exercise rehabilitation group will be required to submit weekly practice logs reporting their training completion, experience, and any discomfort. Participants may submit these practice logs in written form via email, text message, or instant messaging apps, depending on their preference and convenience. Alternatively, participants who find it difficult to submit written logs will have the option to report verbally through a scheduled phone call with a designated member of the research team. For each participant, a designated research member will maintain regular contact. If a participant does not submit their weekly report on time, the research team will proactively follow up by phone or instant messaging apps to remind them to submit their practice log. To further promote accountability and adherence, each participant will receive a minimum of two phone check-ins per month.

During these calls, the research team will:

1. Review the participant’s training progress and address any questions about the intervention.
2. Provide coaching tips or practical advice to help overcome any difficulties in following the exercise program.
3. Discuss potential barriers to adherence (e.g., time constraints, technical issues) and offer feasible solutions.
4. Encourage participants to continue practicing regularly and highlight the importance of consistent participation for achieving the expected benefits.

These follow-up communications will be documented in the study records. If a participant repeatedly fails to submit practice logs or shows signs of poor adherence, the research team will provide additional personalized guidance and, if necessary, involve family members to support the participant’s continued engagement.

### 3.11 Harms and adverse events

Participants’ physical conditions will be closely monitored throughout the trial. Any adverse events (AEs) related to the interventions (e.g., falls, muscle strain) will be recorded and reported in accordance with CONSORT guidelines for non-pharmacological trials. Serious adverse events (SAEs), defined as any unexpected medical occurrence that results in death, is life-threatening, or requires hospitalization, will be specifically documented and handled according to ethical requirements. Before each remote session, participants will receive clear safety instructions and will be asked to report any discomfort immediately through the online platform or by phone. If any participant experiences discomfort during a session, the activity will be immediately halted and medical consultation will be arranged promptly. The research team will follow up as needed and provide appropriate advice, including referral for medical care if necessary. All reported adverse events will be reviewed by a designated safety monitor who is independent of the intervention team.

### 3.12 Participant timeline

**Figure 2:**
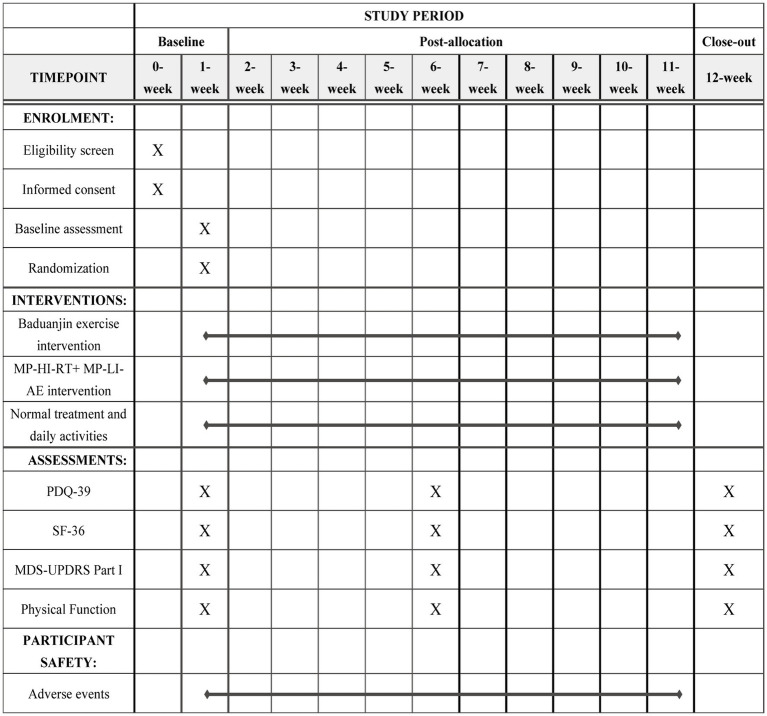
SPIRIT schedule of entrollment, interventions, and assessments.

Figure 2 offers a comprehensive timeline of participant enrollment, intervention implementation, and assessment points across the study period. Assessments will be conducted at baseline (Week 1), interim (Week 6), and final (Week 12).

### 3.13 Outcomes

All outcomes will be measured and documented at baseline assessment (Week 1), interim assessment (Week 6), and final assessment (Week 12). The primary outcomes are quality of life, assessed using the Parkinson’s Disease Questionnaire-39 (PDQ-39), the MOS 36-item Short Form Health Survey (SF-36), and the Unified Parkinson’s Disease Rating Scale developed by the Movement Disorder Society Part I (MDS-UPDRS Part I). The second indicator is physical function, which covers Reaction time, Balance, Physical coordination, Flexibility, and Walking capacity.

### 3.14 Quality of life (QOL)

#### 3.14.1 PDQ-39

PDQ-39 (24, 25) is a validated questionnaire specifically designed to assess quality of life in PD patients. It has demonstrated high reliability and validity and is widely applied in PD-related research. The PDQ-39 encompasses eight domains: mobility, activities of daily living (ADL), emotional well-being, stigma, social support, cognitive impairment, communication, and bodily discomfort.

#### 3.14.2 SF-36

SF-36 (26) is a widely used tool for assessing health status and has been validated across multiple disease populations with strong reliability and validity. It comprises eight dimensions: physical functioning (PF), role physical (RP), bodily pain (BP), general health (GH), vitality (VT), social functioning (SF), role emotional (RE), and mental health (MH).

#### 3.14.3 MDS-UPDRS part I

MDS-UPDRS represents one of the most widely used tools to assess PD progression (27). MDS-UPDRS Part I will be used in this study to assess non-motor experiences of daily living in individuals with PD. Part I consists of patient-reported items evaluating symptoms such as cognitive impairment, mood disturbances (e.g., depression, anxiety), sleep problems, pain, urinary issues, and fatigue. Higher scores indicate greater severity of non-motor symptoms. MDS-UPDRS Part I has demonstrated high internal consistency (Cronbach’s *α* = 0.85), small floor and ceiling effects (2 and 0%, respectively), and good concurrent validity (correlation with the original UPDRS Part I: r = 0.81, *p* < 0.001) (28). Its inclusion in this study will provide a comprehensive evaluation of the non-motor aspects of patient well-being, complementing the broader quality of life assessments captured by the PDQ-39 and SF-36.

### 3.15 Outcomes of physical function

#### 3.15.1 Reaction time

Participants will stand in a natural posture, with a signal light positioned approximately 1 meter in front of their abdomen. Upon illumination of the light, participants responded by bending their knees and jumping upward. A stopwatch will record the reaction time (s), defined as the duration between light activation and the moment the participant jumped. The test will be conducted twice, and the average score will be recorded.

#### 3.15.2 Balance

##### 3.15.2.1 One-legged blind balance test

The one-legged blind balance test will be used to evaluate static balance ability. Participants will stand in a natural posture, and upon hearing the “start” command, close their eyes and lift one foot. The test ended when the supporting foot moved, the lifted foot touched the ground, or the participant opened their eyes. Both the left and right foot will be tested twice, and the average duration (s) of static balance will be recorded using a stopwatch.

##### 3.15.2.2 Y-balance test (YBT)

The Y-Balance Test (YBT) will be utilised to evaluate dynamic balance ability. Participants will stand at the center of a Y-shaped testing apparatus, balancing on one leg while reaching with the other leg in three directions: anterior (forward), posterolateral (backward and outward), and posteromedial (backward and inward). Participants will be required to keep their support foot stable during the test and extend the reaching leg in a controlled manner. The maximum reach distance (cm) for each direction will be recorded. Each leg will be tested twice, with the best attempt noted.

#### 3.15.3 Physical coordination

##### 3.15.3.1 Five times sit-to-stand test (FTSTS)

The FTSTS measured lower limb strength and coordination. Participants began in a seated position on an armless chair, backs against the upright backrest, arms crossed over the chest. Upon the “start” command, participants stand up fully and sit back down five times as quickly as possible. Timing will start when their backs lift off the chair and end when their buttocks touch the seat after the fifth repetition. FTSTS has high inter-rater reliability and test–retest reliability, with intraclass correlation coefficients (ICC) of 0.99 and 0.76, respectively. The average FTSTS completion time was 20.25 ± 14.12 s (29).

##### 3.15.3.2 Timed up and go (TUG)

The TUG test assessed dynamic coordination, gait stability, and fall risk. The test will require an armrest-equipped chair, a stopwatch, and a 3-meter course marked with a thick colored line. Participants will wear their usual footwear, sit with their back against the chair, and place their hands on the armrests. Upon the “start” command, participants will stand up firmly, walk 3 meters as quickly as possible, turn around after crossing the line, walk back to the chair, turn around again, and sit down. The total time (s) taken to complete the task will be recorded.

#### 3.15.4 Flexibility

##### 3.15.4.1 Sit-and-reach test

The Sit-and-Reach Test assessed lower body flexibility. Participants will remove their shoes, sit on a flat surface, extend their legs forward with knees straight, and place their feet slightly apart against a measurement board. With one hand placed over the other, they will gradually reach forward. The farthest fingertip reach (cm) will be recorded, with positive values indicating a reach beyond the feet and negative values indicating a reach falling short of the toes.

##### 3.15.4.2 Back scratch test

The Back Scratch Test evaluated upper limb flexibility and shoulder mobility. Participants will attempt to reach one hand over the shoulder while simultaneously bringing the other hand up from behind the back, trying to touch the fingertips of the opposite hand. The distance between the middle fingers (cm) will be recorded, where 0 cm indicates fingertips just touching, negative values represent a gap between fingertips (e.g., −5 cm), and positive values indicate overlapping fingers (e.g., +3 cm). This assessment will be executed twice with each hand in an alternating sequence. The ultimate score, measured in centimetres (cm), will be computed as the average of the optimal attempts from both arms. If participants experience pain, the test will be immediately discontinued.

#### 3.15.5 Walking capacity

##### 3.15.5.1 Ten-meter walk test (10MWT)

The Ten-Meter Walk Test (10MWT) will be used to evaluate gait speed under both comfortable and maximum speed conditions in individuals with PD. A total distance of 10 meters will be marked on the floor, with additional markers placed at 2 meters from the starting point and 2 meters from the ending point to define a 6-meter timed middle section. Participants will be instructed to begin walking from the starting line and continue past the finish line, walking at their normal pace during the first trial and as fast as safely possible during the second trial. Timing will commence as the participant’s leading foot crosses the 2-meter mark and will stop when the leading foot crosses the 8-meter mark, thereby excluding the initial acceleration and final deceleration phases from the timed measurement. Walking speed (m/s) will be calculated by dividing the timed distance by the time taken to complete the 6-meter section. If participants report fatigue, dizziness, or discomfort, the test will be discontinued immediately.

##### 3.15.5.2 Six-minute walk test (6MWT)

The Six-Minute Walk Test (6MWT) is recommended for assessing walking capacity in people with PD by the American Physical Therapy Guidelines (30). Participants will be instructed to walk along a straight, flat 30-meter indoor corridor, turning as needed, for a total of 6 min at a self-selected comfortable pace without running or jogging. Participants may slow down or stop to rest if needed, but will be encouraged to resume walking as soon as possible. Standardized verbal encouragement will be provided each minute (e.g., “You are doing well. Keep up the good work”). The total distance walked in meters within 6 min will be recorded as the test result. The 6MWT has demonstrated convergent validity with energy cost of walking (*r* = 0.655) and excellent test–retest reliability (ICC = 0.95–0.96) in individuals with PD (31, 32). If participants report excessive fatigue, dizziness, chest pain, or other discomforts during the test, it will be immediately discontinued for safety reasons.

### 3.16 Data collection and management

To ensure the accuracy and reliability of data collection, all personnel responsible for data measurement and recording underwent standardized training on operational procedures. Data monitoring will be conducted by independent personnel who are not involved in the intervention of the study. This process will include reviewing all informed consent forms, verifying data integrity, and cross-checking source documents. All data will be collected at three time points (Week 1, Week 6, Week 12) to monitor short-term and long-term effects. Data will be recorded on standardised report forms and transferred to a web-based data management system (REDCap) through multiple secure entries. Any errors identified during data entry will be corrected, with the researcher providing a signature and date for verification. Participants will have the right to withdraw from the study at any time, for any reason. In cases of withdrawal, data already collected will remain in the dataset unless the participant requests removal.

### 3.17 Statistical analysis

A mixed-effect model will be employed to analyze the experimental data, accounting for individual variability. The model will assess the random effect of participants while adjusting for baseline variables. Baseline characteristics will be compared between groups using appropriate statistical tests (e.g., one-way ANOVA for continuous variables, chi-square test for categorical variables) to ensure group equivalence prior to intervention. If any significant baseline differences are found, they will be adjusted for in the subsequent analyses. To account for differences in exercise intensity and total dose between groups, weekly exercise volume (MET-hours/week) will be calculated and included as a covariate in the mixed-effect model. Intergroup comparisons will be conducted without multiple comparison adjustments. The differences in intervention effects among the three groups will be calculated and expressed as effect sizes with 95% confidence intervals (CIs). For outcome analyses, mixed-effect models will be primarily employed to analyze repeated measures across time points. When analyzing between-group differences at individual time points, one-way ANOVA or Kruskal-Wallis test will be applied, depending on data distribution and normality assumptions. Pairwise post-hoc comparisons will apply Bonferroni correction. All statistical analyses will be conducted using SPSS 26.0 and R 4.0.2, with a significance level of *p* < 0.05.

### 3.18 Blind method

Subjects will be randomly assigned to one of three groups (QG, EG, or CG). The details of randomization and allocation concealment are described in Section 3.4.1.

Blinding procedures will be implemented to minimise bias:

1. Participants will be aware of their assigned intervention, but will not be informed about the details of other group interventions since all remote sessions will be delivered via the remote platform.
2. Outcome assessors will be blinded to group allocation to prevent measurement bias during data collection, and statisticians will be blinded during statistical processing until the final analysis stage.
3. Intervention facilitators will only know their specific group assignment and will not be part of data collection or analysis.

### 3.19 Sample size

The sample size estimation was based on expected changes in PDQ-39 total score, assuming a minimum clinically significant difference of 5 points between groups. Using a power of 80% (1-*β* = 0.80) and a significance level of 0.05 (two-tailed), the required sample size was calculated using G*Power 3.1. Given an estimated standard deviation (SD) of 10.5 and an effect size of 0.5, the initial calculation suggested 42 participants per group. Considering a potential 15% dropout rate, the final sample size was increased to 50 participants per group, resulting in a total of 150 participants.

## 4 Discussion

Baduanjin Qigong has been shown to improve both motor and non-motor symptoms in individuals with PD, contributing to better mobility, balance, and mental well-being (20, 22). Unlike conventional aerobic or resistance training, Baduanjin uniquely integrates gentle physical movements with breathing regulation and mental focus, offering potential benefits for managing not only motor impairments but also common non-motor issues such as anxiety, depression, sleep disturbances, and cognitive decline (33, 34). Recent systematic reviews and meta-analyses have confirmed its effectiveness as a comprehensive rehabilitation approach for individuals with PD, highlighting its high acceptability and safety and reporting significant improvements in both physical function and mental health (35, 36).

However, despite promising evidence, few randomized controlled trials have directly compared Baduanjin Qigong with traditional exercise interventions in PD populations. Most existing studies have focused predominantly on motor outcomes, while comprehensive evaluations of psychological well-being and overall quality of life remain limited. Meanwhile, the majority of Baduanjin interventions have traditionally been conducted through in-person sessions. This delivery mode may not fully address the practical barriers faced by PD patients. Traditional face-to-face rehabilitation programs often face challenges such as transportation difficulties, time constraints, and financial burdens, which can limit PD patient participation and increase pressure on healthcare systems (37). Based on this, we have designed a randomized controlled trial comparing remote Baduanjin Qigong with conventional exercise programs, focusing on both quality of life and physical function, to determine whether remote Baduanjin Qigong offers unique advantages over standard exercise modalities in managing PD and can serve as an effective and feasible intervention for individuals with mild to moderate PD.

The development of internet-based remote interventions has created new opportunities for delivering rehabilitation with greater flexibility, lower costs, and broader accessibility, effectively helping patients—particularly those in rural or remote areas—overcome mobility and financial barriers that often hinder participation in conventional in-person programs (38–40). Moreover, remote rehabilitation typically occurs in patients’ familiar home environments, enhancing comfort and enabling more personalized care through telemonitoring and frequent provider interactions, which can improve adherence (41, 42). Nevertheless, conducting rehabilitation at home may reduce patients’ social interactions and limit the emotional connection and support that typically occur in in-person sessions, which could influence motivation and engagement (39). In addition, remote interventions encounter significant challenges, including the digital divide that disproportionately affects older adults with limited digital literacy. Practical issues such as insufficient training in digital skills and difficulties in managing technology also pose barriers for certain patient groups (43). Therefore, considering these limitations, video conferencing has emerged as a highly interactive and user-friendly tool for delivering remote healthcare services to individuals with mild to moderate PD. Its relatively simple technological requirements and intuitive interfaces make it accessible even to those with limited digital experience, while still facilitating real-time guidance and feedback, fostering greater patient engagement and trust in the rehabilitation process (44, 45). To this end, in our study, we plan to adopt video conferencing as a convenient and user-friendly format and will implement specific strategies to address these challenges, including providing pre-session internet literacy training, offering real-time technical support during the live sessions, and engaging family members or caregivers to assist participants who may have difficulties using the technology. Furthermore, recent studies indicate that remote interventions can achieve outcomes compared to those of in-person therapy, significantly improving various motor functions and alleviating non-motor symptoms in PD patients, thereby contributing to better overall quality of life (46–49).

Despite the strengths of our study design, several limitations must be acknowledged. Funding constraints resulted in a relatively short intervention and follow-up period (12 weeks), which may limit capturing long-term effects. Future studies should consider longer follow-up periods and larger sample sizes to assess the sustained benefits of remote Baduanjin Qigong intervention in PD. Additionally, while our study evaluates motor and non-motor improvements, further research is needed to explore whether Baduanjin Qigong can delay PD progression or reduce medication dependency.
